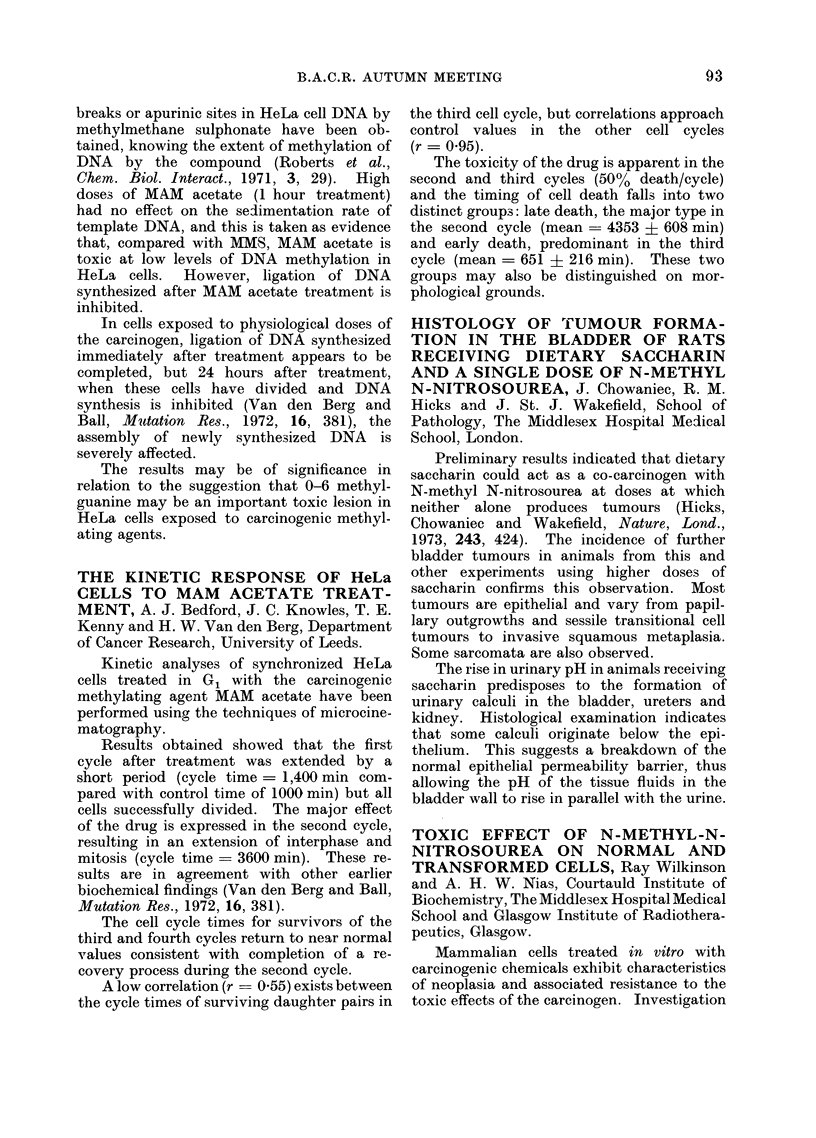# Proceedings: The kinetic response of HeLa cells to MAM acetate treatment.

**DOI:** 10.1038/bjc.1974.17

**Published:** 1974-01

**Authors:** A. J. Bedford, J. D. Knowles, T. E. Kenny, H. W. Van den Berg


**THE KINETIC RESPONSE OF HeLa CELLS TO MAM ACETATE TREATMENT,** A. J. Bedford, J. C. Knowles, T. E. Kenny and H. W. Van den Berg, Department of Cancer Research, University of Leeds.

Kinetic analyses of synchronized HeLa cells treated in $G_1$ with the carcinogenic methylating agent MAM acetate have been performed using the techniques of microcinematography.

Results obtained showed that the first cycle after treatment was extended by a short period (cycle time = 1,400 min compared with control time of 1000 min) but all cells successfully divided. The major effect of the drug is expressed in the second cycle, resulting in an extension of interphase and mitosis (cycle time = 3600 min). These results are in agreement with other earlier biochemical findings (Van den Berg and Ball, *Mutation Res.*, 1972, **16**, 381).

The cell cycle times for survivors of the third and fourth cycles return to near normal values consistent with completion of a recovery process during the second cycle.

A low correlation ($r = 0{\cdot}55$) exists between the cycle times of surviving daughter pairs in the third cell cycle, but correlations approach control values in the other cell cycles ($r = 0{\cdot}95$).

The toxicity of the drug is apparent in the second and third cycles (50% death/cycle) and the timing of cell death falls into two distinct groups: late death, the major type in the second cycle (mean = 4353 ± 608 min) and early death, predominant in the third cycle (mean = 651 ± 216 min). These two groups may also be distinguished on morphological grounds.